# The association of systemic inflammation and cognitive functions of pre-school aged children residing in a *Schistosoma haematobium* endemic area in Zimbabwe

**DOI:** 10.3389/fimmu.2023.1139912

**Published:** 2023-04-18

**Authors:** Maritha Kasambala, Samson Mukaratirwa, Arthur Vengesai, Tariro Mduluza-Jokonya, Luxwell Jokonya, Herald Midzi, Rutendo Birri Makota, Arnold Mutemeri, Emmanuel Maziti, Bazondlile Dube-Marimbe, Dixon Chibanda, Francisca Mutapi, Takafira Mduluza

**Affiliations:** ^1^ School of Life Sciences, University of KwaZulu-Natal, Durban, South Africa; ^2^ Department of Biological Sciences and Ecology, University of Zimbabwe, Harare, Zimbabwe; ^3^ One Health Center for Zoonoses and Tropical Veterinary Medicine, Ross University School of Veterinary Medicine, Basseterre, Saint Kitts and Nevis; ^4^ Department of Biochemistry, Faculty of Medicine and Health Sciences, Midlands State University, Gweru, Zimbabwe; ^5^ Department of Surgery, College of Health Sciences, University of Zimbabwe, Harare, Zimbabwe; ^6^ School of Medicine and Medical Sciences, University of KwaZulu-Natal, Durban, South Africa; ^7^ Department of Biotechnology and Biochemistry, University of Zimbabwe, Harare, Zimbabwe; ^8^ Department of Psychiatry, College of Health Sciences, University of Zimbabwe, Harare, Zimbabwe; ^9^ Ashworth Laboratories, Institute for Immunology and Infection Research and Centre for Immunity, Infection and Evolution, School of Biological Sciences, University of Edinburgh, Edinburgh, United Kingdom

**Keywords:** cognitive functions, cytokines, hematological parameters, pre-school aged children, schistosomiasis, systemic inflammation

## Abstract

**Background:**

Cognitive function is negatively impacted by schistosomiasis and might be caused by systemic inflammation which has been hypothesized to be one of the mechanisms driving cognitive decline, This study explored the association of systemic inflammatory biomarkers; interleukin (IL)-10, IL-6, IL-17, transforming growth factor (TGF-β), tumor necrosis factor (TNF-α), C-reactive protein (CRP) and hematological parameters with cognitive performance of preschool-aged children (PSAC) from an Schistosoma haematobium endemic area

**Methods:**

The Griffith III tool was used to measure the cognitive performance of 136 PSAC. Whole blood and sera were collected and used to quantify levels of IL-10, TNF-α, IL-6, TGF-β, IL-17 A and CRP using the enzyme-linked immunosorbent assay and hematological parameters using the hematology analyzer. Spearman correlation analysis was used to determine the relationship between each inflammatory biomarker and cognitive performance. Multivariate logistic regression analysis was used to determine whether systemic inflammation due to S. haematobium infection affected cognitive performance in PSAC.

**Results:**

Higher levels of TNF-α and IL-6, were correlated with lower performance in the Foundations of Learning domain (r = -0.30; p < 0.001 and r = -0.26; p < 0.001), respectively. Low cognitive performance in the Eye-Hand-Coordination Domain was observed in PSAC with high levels of the following inflammatory biomarkers that showed negative correlations to performance; TNF-α (r = -0.26; p < 0.001), IL-6 (r = -0.29; p < 0.001), IL-10 (r = -0.18; p < 0.04), WBC (r = -0.29; p < 0.001), neutrophils (r = -0.21; p = 0.01) and lymphocytes (r = -0.25; p = 0.003) The General Development Domain correlated with TNF-α (r = -0.28; p < 0.001) and IL-6 (r = -0.30; p < 0.001). TGF-β, L-17A and MXD had no significant correlations to performance in any of the cognitive domains. The overall general development of PSAC was negatively impacted by S. haematobium infections (OR = 7.6; p = 0.008) and (OR = 5.6; p = 0.03) where the PSAC had higher levels of TNF-α and IL-6 respectively.

**Conclusion:**

Systemic inflammation and S. haematobium infections are negatively associated with cognitive function. We recommend the inclusion of PSAC into mass drug treatment programs.

## Introduction

Neurodevelopment is critical during early childhood development which is a time period involving rapid brain development of neurological pathways that influence cognitive functioning that includes memory, attention, language learning, perception, decision making and problem-solving skills (1, 2). Factors such as malnutrition, poverty, inflammation and infectious diseases predispose children, particularly those that reside in resource-poor communities of developing countries to neurodevelopmental delays (3, 4). Schistosomiasis (bilharzia) is an acute and chronic neglected tropical disease caused by *Schistosoma* spp. and has been reported in over 70 countries where 90% of populations requiring treatment reside in the rural areas of sub-Saharan Africa (5, 6). Amongst the negative impacts of schistosomiasis to infected adults and especially children are inflammatory reactions and hematologic changes accompanied with poor cognitive performance (7–11).

To date, there are no studies that have attempted to investigate the mechanism of how schistosomiasis causes cognitive impairment especially in infected children. However, it is widely believed that chronic host systemic immune responses and inflammation to infection from diseases such as schistosomiasis are the major causes of cognitive decline and could be one of the key mechanisms through which schistosomiasis affects cognitive functioning (12). The immunopathology of acute and chronic schistosomiasis is characterized by T helper (Th1) and T helper 2 (Th2) immune responses against the different types of antigens that are released during the growth and reproductive stage of the schistosomes (13). At the onset of infection for 6 weeks, a Th1 immune response involving the production of pro-inflammatory cytokines; TNF-α, IFN-γ, IL-6 and IL-1 induce cell-mediated immunity and phagocytosis (14). IL-4, IL-5, TGF-β, IL-17A and IL-10 (anti-inflammatory cytokine) are Th2 cytokines that downregulate the Th1 response, promote granuloma formation and induce the humoral mediated immunity mostly against the egg antigens (15–17). Some of the cytokines produced during schistosome infections (interferon-gamma (IFN)-λ, TNF-α, IL-10, IL-6, TGF-β, and IL-17A) have been studied in different populations and age groups with different infectious conditions and have been associated with the alteration of cognitive functions and behavioral manifestations (18, 19). The immune cells, which are part of the hematological parameters, are the sources of inflammatory cytokines which play a key role in communicating with the central nervous system (20). Inflammatory mediators such as cytokines induce the permeability of the blood-brain barrier (BBB) which preserves the central nervous system (CNS) (21–23).

Previous studies have focused on the possible impacts of schistosomiasis on cognitive functions in children but only few studies have explored the effects of schistosomiasis on early childhood development. There are no studies to date that have examined the relationship between inflammatory biomarkers (cytokines and hematological parameters) and cognitive performance in populations infected with schistosomes in endemic areas, however, various studies on other diseases are indicative of the complex diverse relationship between different cytokines and cognitive domains. Studies have reported on contradictory results which entail a mixture of associations and no associations between cytokines levels and cognitive function in different domains (24–29).

This study thus aims to provide information that contributes towards understanding how schistosomiasis might be impacting the cognitive functioning in PSAC by exploring the role of systemic inflammation which encompasses the measurement of various immune cells, cytokines and inflammatory proteins in PSAC residing in a urinary schistosomiasis endemic area in Zimbabwe.

## Methods

### Study design and participants

This case control cross sectional study was part of another study that primarily aimed at determining the impacts of schistosomiasis on the early childhood development that was conducted in Murewa, Zimbabwe (30). The main objective of the study was to determine the role of systemic inflammation on cognitive performance by investigating the following two objectives; i) Determining if there were any correlations between inflammatory biomarker levels and cognitive functioning and, ii) Determining which possible explanatory factors could be driving cognitive function in PSAC with significant correlations between inflammatory biomarker levels and cognitive function. PSAC from Zimbabwe (Magaya village in Murewa district) were screened for schistosomiasis. This area has a high prevalence of *S. haematobium* (> 50%). Thirty PSAC were diagnosed with *S. haematobium* infection through urine filtration and 106 non-infected PSAC from non-permanent residents of Magaya village were selected as controls.

*Inclusion and exclusion criteria*. PSAC who were eligible for recruitment into the study met the following inclusion criteria; (i) were lifelong occupants of the study area (Murewa, Zimbabwe) (ii) had never received anthelminthic medication (iii) were aged less than 72 months (iv) provided 3 urine samples for the diagnosis of *S. haematobium* and stool samples for soil transmitted helminths and *S. mansoni* diagnosis (v) written consent obtained from parent to participate in the study.

Children were excluded in the study if; (i) They had mental or physical disabilities such as attention-deficit disorder which predisposes them to attaining low cognitive scores, (ii) had a positive diagnosis of soil transmitted helminths and/or *S. mansoni.*

Sample size calculation was done using the assumption of 50% exposure in cases and 22% exposure in controls and an Odds Ratio of 3.5 to report a power of 80% and 95% confidence interval.

### Parasitological examination

Parasitological screening for *S. haematobium*, soil transmitted helminths and *S. mansoni* eggs was conducted as described previously (30). Each PSAC provided at least 3 urine and stool samples over 3 cumulative days. Urine samples were used to microscopically screen for *S. haematobium* eggs using the urine filtration method (31) whilst *S. mansoni* and soil transmitted helminths were screened from the stool samples using the Kato-Katz method (32).

### Nutritional status

Nutritional status was scored using the WHO child growth standards from the WHO Anthro software, version 3.0.1(http://www.who.int/childgrowth/en/) as described in a previous study (33). Briefly, measurements of height, weight and the mid upper arm circumference (MUAC) were taken from PSAC using a stadiometer (Gima^®^), electronic scale (Gima^®^) and MUAC tape (AnthroFlex^®^) respectively. These measurements were used to obtain Z-scores and PSAC with BMI Z-scores < -2 were classified as having a low nutritional status.

### Analysis of cognitive performance

Cognitive performance measures of early childhood development was determined using the Griffiths III psychometric tool which was administered by a psychologist on the same day that blood samples were collected. This tool measures 5 cognitive subscales whose average developmental quotient scores give the general development profile of a child (general quotient score). The 5 cognitive subscales that were assessed include; The Foundations of Learning, Language and Communication, Eye and Hand Coordination, Personal-Social-Emotional and Gross Motor function. The Foundations of learning subscale assesses learning during the early childhood period. The Language and Communication subscale is used to assess expressive and receptive language including the child’s ability to communicate. The Eye and Hand Coordination subscale measures the child’s small muscle coordination of eyes with hands. The Personal-Social-Emotional subscale is used to assess the child’s ability to manage emotions, peer relationships and how they articulate to tasks and the Gross Motor function which assesses a child’s development in the areas of physical skills development. Developmental Quotient (DQ) scores >88 was used to classify cognitive performance in PSAC as normal from each Griffith Mental Developmental subscale (34).

### Blood collection and the determination of inflammatory biomarker levels

Blood samples (5ml) were collected from all the PSAC on the same day and prior to the administration of the cognitive assessment by a clinician. The serum samples were used for the determination of cytokines (IL-6, TNF-α, TGF-β, IL-10 and IL-17A) and CRP. CRP levels were measured using a commercial Duoset Enzyme-Linked Immunosorbent Assay (ELISA) kit (R & D Systems, Minneapolis, MN, USA) according to the protocol provided by the manufacturer. The cytokine levels of IL-6, TNF-α, TGF-β, IL-10 and IL-17A were measured using the ELISA according to the manufacturer’s (Mabtech Company, Stockholm, Sweden) protocol. Briefly, serum samples for each inflammatory biomarker were run in duplicates on the same microtiter plate. Standards of known concentration were used to determine the concentrations of the cytokines. The Dynatech Immulon^®^ microtiter plates were coated overnight with the coating solution (capture monoclonal antibody mixed with phosphate-buffered saline (PBS)). The microtiter plates were washed and blocked with the Tris- buffered saline (TBS) and were incubated for 30 minutes. After another wash step, the serum samples that were diluted in the reagent diluent were micro pipetted into the microtiter plates and were incubated at 37°C for 2 hours. After washing the plates, the conjugated antibody was added. The microtiter plates were incubated for an hour after which the substrate o-phenylenediamine dihydrochloride (OPD) was added. The microtiter plates were incubated for 20 minutes in dark conditions. The optical densities were immediately measured at 450nm using a microplate reader (Micro read 1000).

### Hematological analysis and the determination of CRP levels

The determination of total white blood cells (WBC), hemoglobin (HGB), lymphocyte (LYM), Mixed Cell Count (MXD) and neutrophil count was done using a hematology analyzer (MaxM; Coulter, Fullerton, CA) from whole blood samples that were in tubes containing ethylene diamine tetra acetic acid (EDTA).

### Statistical analyses

The data obtained was checked for normality and analyzed using Stata Version 17.0 (StataCorp LLC, Texas, USA). Wilcoxon rank sum was used to determine the differences in cytokine concentration levels by cognitive status, by infection status and the differences in cognitive performance by infection status. The Spearman correlation was used to assess the relationship between the cytokine levels and cognitive performance in the cognitive subscales. Inflammatory biomarkers that were found to have significant correlations with cognitive performance were considered as predictors to cognitive function. PSAC who had higher levels of the significant inflammatory biomarkers than other PSAC were used for multivariate regression analysis. The multivariate regression analysis was done to determine the adjusted odds of *S. haematobium* infection having an association with cognitive performance after adjusting for possible explanatory variables (age, sex, hemoglobin concentrations and nutritional status) which were used as continuous variables. P-values < 0.05 were considered as significant.

## Results

### Demographic characteristics of study participants

**Table 1** summarizes the characteristics of the PSAC who took part in this study, the prevalence *S. haematobium* infection among the study participants was 22.06% and the mean egg count/10ml of urine was 13.5. There was a higher prevalence of *S. haematobium* infection in female PSAC (73%) than in male PSAC (27%). No *S. mansoni* or soil-transmitted helminth eggs were detected in the study population. Out of the entire population, 53 PSAC (39%) did not have toilets in their households, while 40 PSAC (30%) had toilets and 21% did not respond on the presence or absence of a toilet in their households. Normal performance across the cognitive domains was observed from the majority of the PSAC.

**Table 1:** Characteristics of preschool aged children who took part in the study.

Variable	Whole cohort (n=136)	Uninfected	Infected	P- value
BMI Z-score Median (IQR)	-0.63 (1.26)	-0.66 (-1.2)	-0.32 (-1.00)	0.3808
Age (months) Median (IQR)	51 (23)	50 (25)	52 (20)	0.0803
*S. haematobium* infection mean eggs/10ml urine (SD)		0	13.5(28.5)	
Sex:				
Female (%)	63	38.68	73.3	
Male (%)	73	61.32	26.67	**0.001**
Cognition Domains:	Whole cohort (n=136)	Normal performance	Low Performance	P- value
Foundations of Learning n (%)		89 (65)	47 (35)	**0.001**
Language and Communication n (%)		80 (59)	56 (41)	**0.001**
Eye-Hand `Coordination n (%)		112 (82)	24 (18)	**0.001**
Personal-Social-Emotional n (%)		124 (91)	12 (9)	**0.001**
Gross Motor Function n (%)		130 (96)	6 (4)	**0.001**
Overall General Development n (%)		121 (89)	15 (11)	**0.001**

SD, standard deviation; IQR, interquartile range; g/dl, grams per deciliter. P-value in bold signifies significance at P < 0.05.

### Inflammatory biomarker profiles of study participants

**Figure 1:**
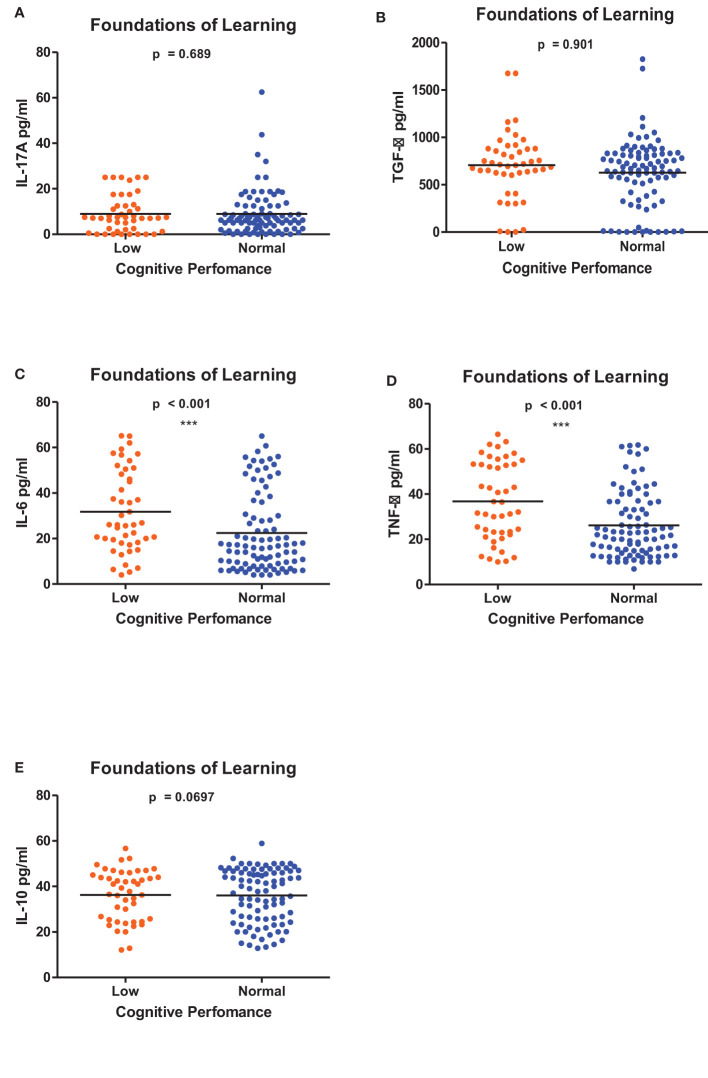
**(A-E)** Cytokine concentration level comparisons between low and normal PSAC performers in the Foundations for Learning Domain using the Wilcoxon matched-pairs signed rank test. ** = p ≤ 0.01.

**Figure 2:**
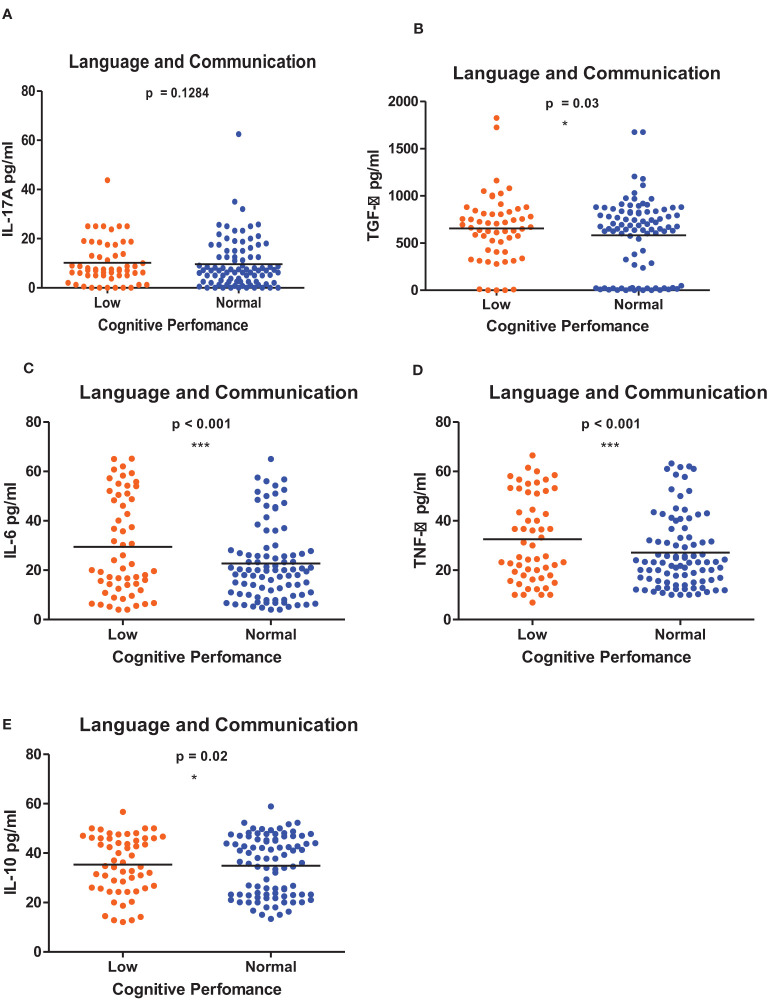
**(A-E)** Cytokine concentration level comparisons between low and normal PSAC performers in the Language and Communication Domain using the Wilcoxon matched-pairs signed rank test. * = p ≤ 0.05, *** = p ≤ 0.001.

**Figure 3:**
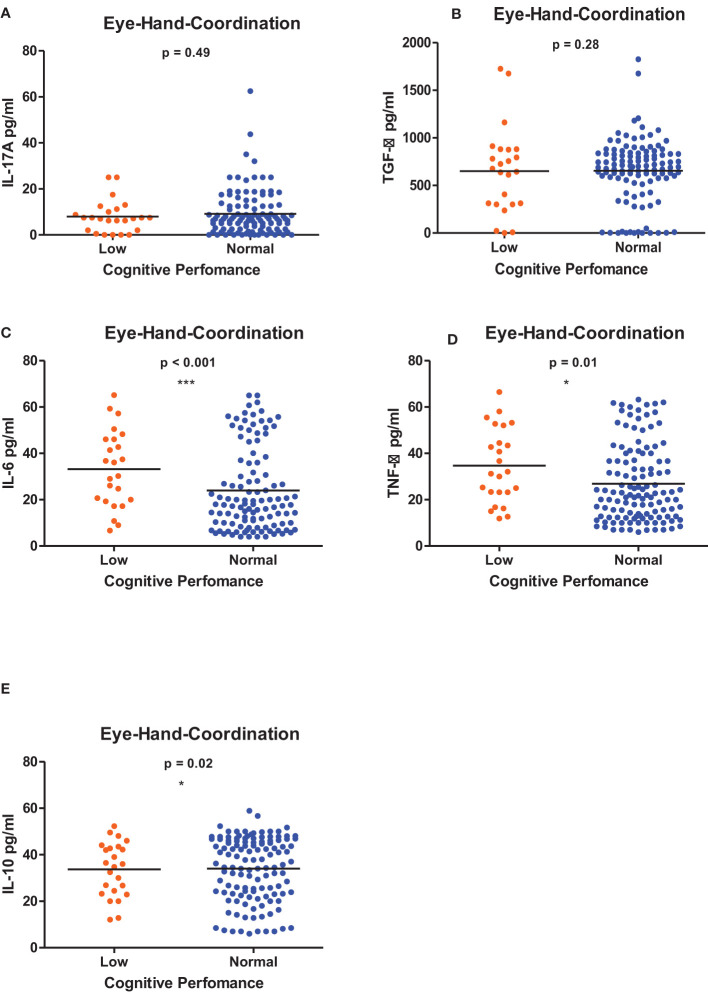
**(A-E)** Cytokine concentration level comparisons between low and normal PSAC performers in the Eye-Hand-Coordination Domain using the Wilcoxon matched-pairs signed rank test. * = p ≤ 0.05, *** = p ≤ 0.001.

**Figure 4:**
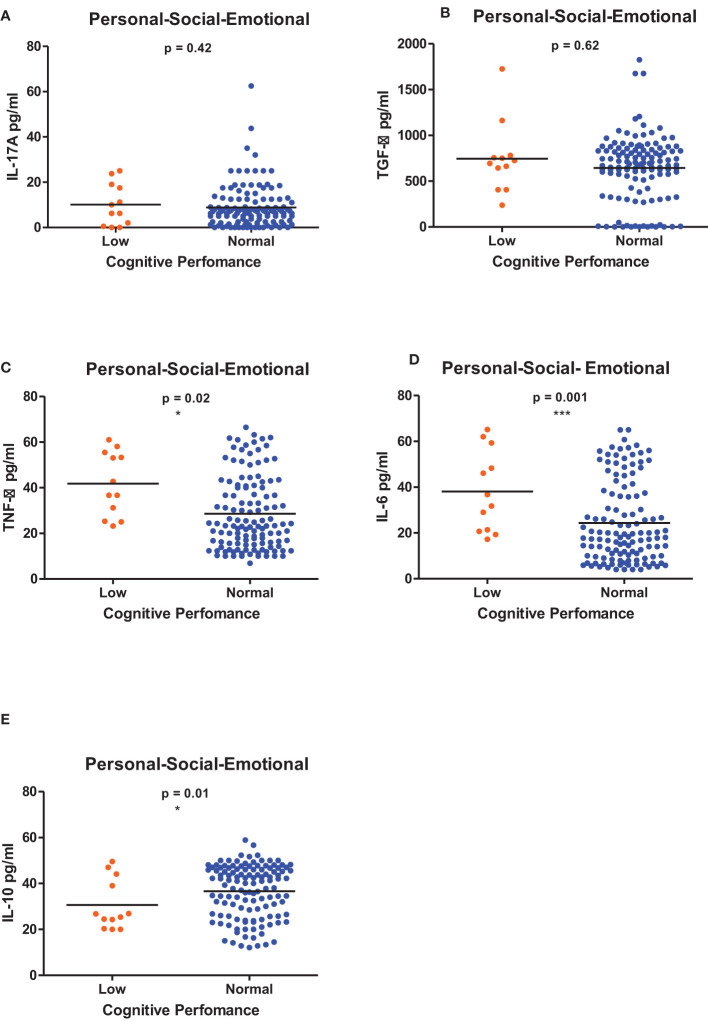
**(A-E)** Cytokine concentration level comparisons between low and normal PSAC performers in the Personal-Social-Emotional Domain using the Wilcoxon matched-pairs signed rank test. * = p ≤ 0.05, *** = p ≤ 0.001.

**Figure 5:**
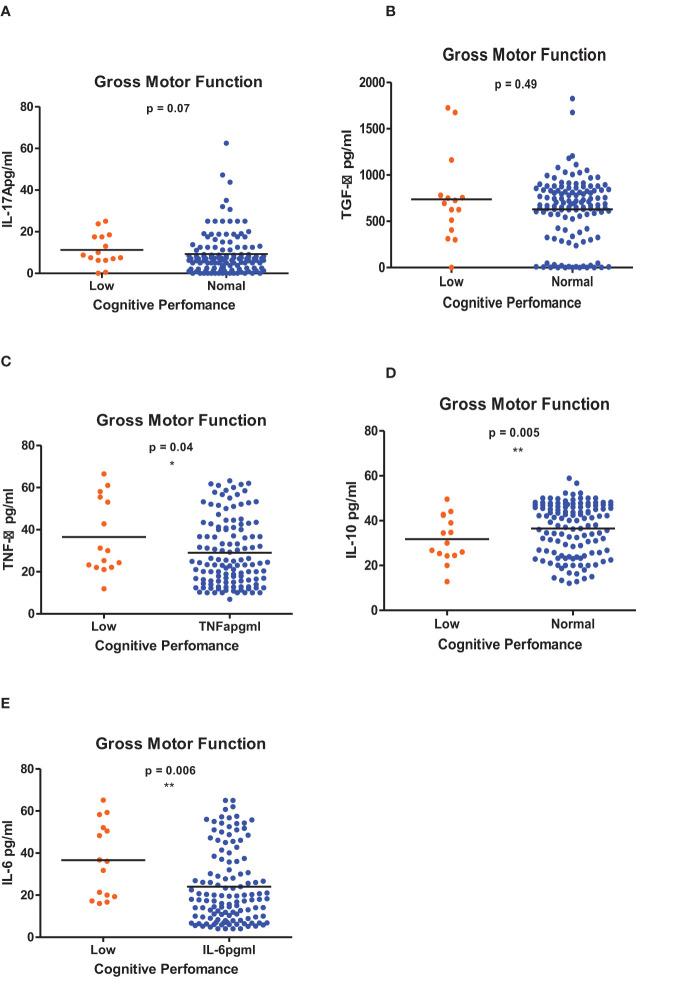
**(A-E)** Cytokine concentration level comparisons between low and normal PSAC performers in the Gross Motor Function Domain using the Wilcoxon matched-pairs signed rank test. * = p ≤ 0.05, ** = p ≤ 0.01.

**Figure 6:**
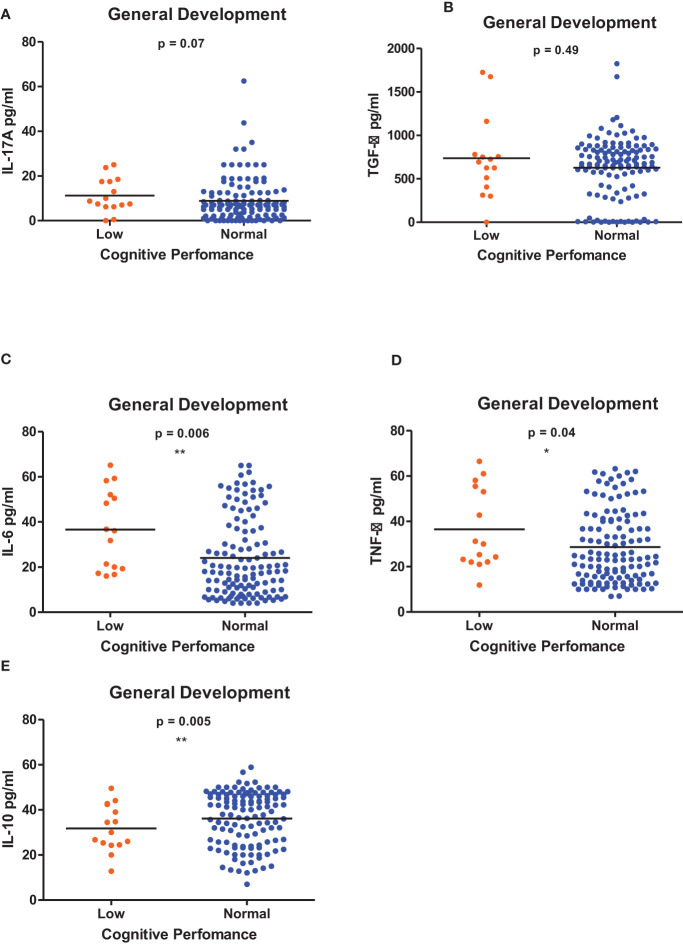
**(A-E)** Cytokine concentration level comparisons between low and normal PSAC performers in the General Development Domain using the Wilcoxon matched-pairs signed rank test. * = p ≤ 0.05, ** = p ≤ 0.01.

**Figures 1**–**6** summarizes the comparison between inflammatory biomarker levels and cognitive performance. PSAC who were categorized as low performers in the Foundations of Learning subscale (**Figures 1A-E**) had significantly higher levels of IL-6 (p < 0.001**)** and TNF-α (p = 0.007**)**. There was no significant difference observed in the cytokine levels of IL-17A (p = 0.80), TGF-β (p = 0.21) and IL-10 (p = 0.89) between PSAC who were low and normal performers in the Foundations of Learning Domain. In the Language and Communication Domain (**Figures 2A-E**), there were significant differences in the inflammatory markers levels of TGF-β, IL-6, TNF-α and IL-10 between low and normal performers while there was no significant difference in performance amongst the inflammatory marker levels of IL-17A. Significant differences in IL-6, TNF-α and IL-10 levels were observed between low and normal PSAC performers in the Eye-Hand-Coordination (**Figures 3C-E**), in the Personal-Social-Emotional Domain (**Figures 4C-E**) and in the General Development Domain (**Figures 6C-E**).

### Correlations between inflammatory biomarkers and cognitive performance

Significant correlations were evident between some of the inflammatory biomarkers (TNF-α, IL-6, IL-10, CRP, HGB, WBC, neutrophils and lymphocytes) and cognitive performance in various cognitive domains in the study participants (**Table 2**). High TNF-α concentration levels were correlated to low performance in the Foundations of Learning subscale (r = -0.30; p < 0.001), Eye-Hand-Coordination (r = -0.26; p < 0.001), General Development Domain (r = -0.28; p < 0.001). IL-6 concentration levels were also negatively correlated to cognitive performance in the Foundations of Learning (r = -0.33; p < 0.001), Eye-Hand-Coordination (r = -0.29; p < 0.001) and General Development Domain (r = -0.30; p < 0.001) suggesting that an increase in IL-6 concentration levels resulted in lower cognitive performance. Higher levels of IL-10 concentration were correlated to better performance in the Eye-Hand-Coordination (r = -0.18; p < 0.04). CRP levels were negatively correlated to performance in the Foundation of Learning Domain (r = - 0.26, p = 0.008). This indicated that increased CRP levels were associated with lower performance in the Foundation of Learning Domain.

**Table 2:** Spearman Correlation analysis of inflammatory biomarkers and cognitive performance of preschool aged children.

Inflammation Biomarker	Foundations of Learning		Language and Communication		Eye-Hand Coordination		Personal-Social-Emotional		Gross Motor Function		General Development	
N = 136	R	p	R	p	R	p	R	p	R	p	R	p
TNF-α	**-0.30**	**0.001**	-0.11	0.19	**-0.26**	**0.001**	-0.05	0.60	-0.14	0.10	**-0.28**	**0.001**
IL-6	**-0.33**	**0.001**	-0.13	0.12	**-0.30**	**0.001**	-0.13	0.13	-0.10	0.27	**-0.30**	**0.001**
IL-10	0.05	0.57	-0.01	0.93	**0.18**	**0.04**	0.003	0.97	0.14	0.11	0.04	0.65
IL-17	0.65	0.88	-0.12	0.15	-0.09	0.32	0.02	0.85	0.16	0.06	-0.07	0.46
TGF-β	-0.06	0.48	-0.01	0.91	0.01	0.93	-0.15	0.08	0.08	0.34	-0.09	0.30
CRP	**-0.28**	**0.008**	-0.02	0.85	-0.16	0.07	0.05	0.53	0.02	0.79	-0.14	0.09
Neutrophils	-0.08	0.35	-0.10	0.27	**-0.21**	**0.01**	-0.12	0.17	-0.06	0.47	-0.14	0.09
Lymphocytes	-0.01	0.87	-0.16	0.06	**-0.25**	**0.003**	0.05	0.53	-0.08	0.36	-0.14	0.09
WBC	-0.05	0.55	-0.14	0.12	**-0.29**	**<0.001**	0.01	0.88	-0.06	0.51	-0.14	0.09
MXD	0.10	0.23	0.16	0.07	0.15	0.08	0.02	0.82	0.06	0.52	0.14	0.12
NLR	-0.08	0.36	0.04	0.63	0.03	0.71	-0.14	0.11	0.02	0.80	-0.01	0.91

TNF- α, tumor necrosis factor α; IL-6, interleukin 6; IL-10, interleukin 10; IL-17, interleukin 17; TGF-β, transforming growth factor; WBC, white blood cell count; CRP, C-reactive protein. WBC, white blood cell count; MXD, mixed cell count (monocytes, basophils and eosinophils); NLR, Neutrophil-to-lymphocyte ratio; R-Correlation coefficient, p-value in bold signifies significance at P < 0.05.

WBC, neutrophils and lymphocytes were negatively correlated to cognitive performance in the Eye-Hand-Coordination Domain (r = -0.29; p < 0.001), (r = -0.21; p = 0.01) and (r = -0.25; p = 0.003 respectively). This indicates that higher levels of WBC, neutrophils and lymphocytes were associated with lower performance in the Eye-Hand-Coordination Domain. The other inflammatory biomarkers (TGF-β, L-17A and MXD had no significant correlations to performance in any of the cognitive domains.

### Multivariate logistic regression analysis of cognitive performance

Multivariate logistic regression was done for the inflammatory biomarker variables (TNF-α, IL-6, IL-10, CRP, HGB, WBC, MCHC, neutrophils and lymphocytes). For each inflammatory biomarker variable group, PSAC were categorized as having either high or low inflammatory biomarkers levels. PSAC with inflammatory biomarker levels that had non-significant correlations with cognitive performance were excluded from their respective multivariate logistic regression analysis.

### Foundations of learning domain

**Table 3** summarizes the multivariate analysis of cognitive function in the Foundations of Learning Domain in PSAC and the inflammatory biomarker variables (IL-6, TNF-α and CRP). There was no significant associations observed between performance in this domain and the possible explanatory variables (sex, hemoglobin, age, nutrition status and *S. haematobium* infection status) in PSAC who were categorized as having higher IL-6. *Schistosoma haematobium* infection status had significant greater odds of low performance in the Foundations of Learning Domain in PSAC who were categorized as having high TNF-α. This implies that PSAC who had *S. haematobium* infections were 2.8 times higher chances of performing lower in comparison to PSAC who were uninfected. There were no significant associations observed in the other explanatory variables (sex, nutritional status, hemoglobin levels and age).

**Table 3:** Multivariate regression analysis of cognitive domains and explanatory variables.

Inflammatory biomarker and Cognitive Domain	Sex	Nutritional Status	Hemoglobin	Age	*S. haematobium* Infection status
	AOR (95% CI) p value	AOR (95% CI) p value	AOR (95% CI) p value	AOR (95% CI) p value	AOR (95% CI) p value
TNF-α (n = 128)					
FLDQ	1.3 (0.6-2.8) 0.588	0.4 (0.04-3.8) 0.423	1.2 (0.9-1.6) 0.317	1 (0.9-1) 0.07	**2.8 (1.1-7.1)** **0.02**
EHCDQ	**3.6 (1-11.7)** **0.03**	1.4 (0.1-15) 0.786	1.2 (0.9-1.8) 0.309	0.9 (0.9-1) **0.001**	**5.9 (1.6-21)** **0.006**
GDDQ	1 (0.3-4.2) 0.922	2.1 (0.2-25.5) 0.545	0.7 (0.4-1.2) 0.162	**1 (0.9-1)** **0.03**	**7.6 (1.7-34)** **0.008**
IL-6 (n = 73)					
FLDQ	1.3 (0.6-3.2) 0.509	0.6 (0.1-7) 0.693	1.1 (0.8-1.5) 0.560	0.9 (0.9-1.0) 0.06	2.4 (0.9-6.5) 0.08
EHCDQ	4.8 (1.4-16.9) 0.01	2.5 (0.2-35.6) 0.509	1.3 (0.9-1.9) 0.236	**0.9 (0.9-1)** **0.005**	**4.8 (1.3-18.2)** **0.02**
GDDQ	1.1 (0.3-4.3) 0.879	2.9 (0.2-41.4) 0.424	0.6 (0.4-1) 0.102	**0.9 (0.9-1)** **0.02**	**5.6 (1.2 -25.5)** **0.03**
CRP (n = 136)					
FLDQ	12 (0.5-2.5) 0.697	1 (0.2-5.6) 0.956	1 (0.9-1) 0.302	1 (0.9-1.7) 0.075	**2.8** (**1-7**) **0.024**
IL-10 (n = 136)					
EHCDQ	**4 (1.9-24)** **0.003**	1.4(0.1-14.8) 0.787	1.2 (0.8-1.8) 0.304	**0.9 (0.9-1)** **0.001**	**6.8 (1.9-24)** **0.003**
Neutrophils (n = 120)					
EHCDQ	**4.2 (1.2-14)** **0.02**	1.3 (0.1-14) 0.834	1.2 (0.7- 1.9) 0.479	**0.9 (0.9-1)** **0.002**	**5.4 (1.4-20)** **0.012**
Lymphocytes (n = 87)					
EHCDQ	**48 (1.2-18.4)** **0.02**	1.4 (0.1-20.1) 0.785	1.3 (0.9-2) 0.171	**0.9 (0.9-1)** **0.006**	**9 (1.8-46.6)** **0.008**
WBC (n = 61)					
EHCDQ	4.3 (0.9-20) 0.06	3.4 (0.2-6.1) 0.408	1.3 (0.8-2.3) 0.325	**0.9 (0.9-1)** **0.02**	5 (0.9-30) 0.08

FLDQ, Foundations of Leaning Development Quotient; EHCDQ, Eye and Hand Coordination Development Quotient; GDDQ, General Development Quotient;. TNF- α, tumor necrosis factor α; IL-6, interleukin 6; IL-10, interleukin 10; WBC, white blood cell count; CRP, C-reactive protein; AOR, adjusted odds ratio; CI, confidence interval; All AORs quantify the odds of poor performance on cognitive domains adjusted for the effect of explanatory variables (sex, nutritional status, hemoglobin level and age). Statistical significance is indicated by bold numbers. Reference categories for the sex category are females and for the S. haematobium infection category are uninfected PSAC. The cut off points used to categorize PSAC as having higher inflammatory biomarkers were TNF- α, IL-6 and IL-10 = 10 pg/ml, CRP = 0.5 mg/ml (35, 36), WBC = 6000/µL, neutrophils = 1500/µL, lymphocytes = 3000/µL (37).

PSAC with high CRP levels and with *S. haematobium* infections were at greater odds 2.8 of lower performance in the Foundations of Learning Domain There were no significant associations between cognitive performance and the explanatory variables (sex, nutritional status, age, and hemoglobin levels) within PSAC who had high CRP levels.

### Eye-hand-coordination domain

Multivariate logistic regression was done for the inflammatory biomarkers (IL-6, TNF-α, IL-10, Neutrophils, Lymphocytes and WBC). Sex, age and *S. haematobium* infection status had significant odds of performance in the Eye-Hand-Coordination Domain in PSAC with high IL-6 levels **(** **Table 3** **)**. Female PSAC were 4.8 times more likely to have better scores than male PSAC. Younger PSAC were more likely to have lower scores than older PSAC. PSAC with *S. haematobium* infections had 4.8 odds of low performance in the Eye-Hand-Coordination Domain compared to uninfected PSAC. *Schistosoma haematobium* infection status, gender and age had significant odds of performance in the Eye-Hand-Coordination in PSAC who had high TNF –α levels. Older PSAC performed better than the younger PSAC. Female PSAC were 3.6 times more likely to have better scores than male PSAC. Infected PSAC had 5.9 odds of lower performance than uninfected PSAC.

In PSAC who had high IL-10 levels, age, gender and *S. haematobium* infection status were found to be associated with performance in the Eye-Hand-Coordination Domain. Younger PSAC performed lower than the older PSAC. Female PSAC had greater odds of better scores than male PSAC and infected PSAC were 6.8 times likely to have lower scores. In PSAC with high lymphocyte levels, sex, age and *S. haematobium* infection status had significant odds of cognitive performance. Male PSAC were 6 times likely to have lower performance than female PSAC. Infected PSAC were 9 times likely to have lower scores than uninfected PSAC while the younger PSAC had greater odds of low performance than the older PSAC. Age, sex and *S. haematobium* infection status were significant factors affecting performance in PSAC who had higher levels of neutrophils and lymphocyte counts in the Eye-Hand-Coordination Domain.

### General development domain

Multivariate logistic regression analysis in **Table 3** indicates that both age and *S. haematobium* infection status had significant odds of performance in the General Development Domain in PSAC who had higher TNF-α and IL-6 levels. Infected PSAC had greater odds of having lower performance scores in comparison to uninfected PSAC. Younger PSAC had lower scores than the older PSAC.

### Inflammatory biomarker profiles of *S. haematobium* infected and uninfected PSAC

**Figure 7:**
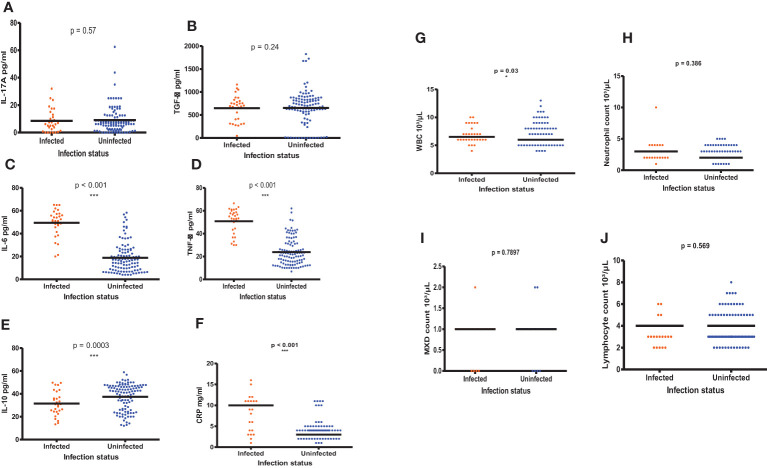
**(A-J)** Comparisons of the inflammatory biomarker levels in infected and uninfected PSAC using the Wilcoxon matched-pairs signed rank test. * = p ≤ 0.05, *** = p ≤ 0.001.

**Figures 7A-J** shows the comparisons of the inflammatory biomarker profiles between *S. haematobium* infected and uninfected PSAC. The levels of IL-17A (**Figure 7A**) and TGF-β **Figure 7B**) were comparable in both infected and uninfected PSAC. Infected PSAC had significantly higher concentration of IL-6 (**Figure 7C**) and TNF-α (**Figure 7D**) in comparison to uninfected PSAC while the levels of IL-10 were higher in the uninfected PSAC group (**Figure 7E**).

There were significant higher WBC counts (p = 0.03, Wilcoxon- test) and MXD (p **<** 0.001, Wilcoxon- test) in PSAC with *S. haematobium* infections compared to the uninfected PSAC. The CRP levels (p **<** 0.001) and NLR (p = 0.03, Wilcoxon- test) were also high in infected PSAC while the MXD were surprisingly high in uninfected PSAC (p = <0.001, Wilcoxon- test). The other hematological parameters (neutrophils and lymphocytes) did not significantly differ between infected and uninfected PSAC.

## Discussion

There is a paucity of studies that have explored the relationship between systemic inflammation and cognition in children and the few existing studies have contradictory and inconclusive results (38–43). Our study is the first to date that has attempted to explore the relationship between ten systemic inflammatory biomarkers and cognitive performance in PSAC during schistosomiasis infections. We report two key findings; the complex relationship between inflammatory biomarkers and reduced cognitive performance as well as the varied negative effects of *S. haematobium* infections on performance in the cognitive domains.

Significant correlations of high levels of systemic inflammatory biomarkers (TNF-α, IL-6, IL-10, CRP, HGB, WBC, neutrophils and lymphocytes) with lower performance in various cognitive domains (Foundations of Learning, Language and Communication, Eye-Hand-Coordination and General Development Domain) were observed in our study and this in agreement with previous studies (44–48). The cognitive performance in the PSAC from our study infected with *S. haematobium* could have been affected by the interaction of systemic cytokines with the central nervous system through the hypothesized routes which include the interaction of systemic cytokines with the receptors on the blood-brain barrier, passage *via* the neural afferent pathway, and/or the circumventricular organs through perivascular spaces (49). Consequently, their passage triggers the brain immune cells (microglia and astrocytes) to produce cytokines that modulate neuronal responses and learning (50–52).

Contrary to our results, IL-6 concentrations were found not to affect cognitive performance in other studies (53, 54). We also found no significant associations between IL-17A concentration and performance in any of the cognitive domains contrary to a study in patients with breast cancer who had lower IL-17A levels which were associated with better performance in the Psychomotor/Gross Motor Function and memory domain (55). We observed the absence of correlations between either of the inflammatory markers TGF-β and IL-17A and cognitive performance and this was likely due to the PSAC having undetectable low levels of TGF-β and IL-17A.

Discrepancies in the results from studies that have investigated the relationship between systemic inflammatory markers and cognitive performance could be because of the differences in study design, study participants, age, the use of different cognitive tools, different timeframes of blood collection, cognitive assessment and quantification of inflammatory markers. In our study, we reduced the risk of bias by ensuring that blood samples were collected on the same day that the cognitive assessments were done, PSAC with schistosomiasis (cases) were matched and compared to PSAC without schistosomiasis (controls), we controlled for the most common confounding factors of cognitive performance and the production of inflammatory biomarkers.

After analysis of the potential explanatory variables that could be influencing cognitive performance and the inflammatory biomarker concentrations, our results indicate that although hemoglobin levels and nutritional status did not have any relationship with cognitive performance, age and gender were found to influence cognitive function which was in agreement to results from previous studies (56, 57). This further emphasizes the importance of controlling for the common potential confounding factors during the analysis of results from cognition based studies as cognitive performance is affected by various factors (8). We also report comparable results of significant greater odds of low performance in PSAC with *S. haematobium* infection in comparison to uninfected PSAC in the Foundations of Learning, Eye-Hand Coordination and General Development Domain (11, 58).

Furthermore, there were significant differences between infected and uninfected PSAC’s TNF-α, IL-6, WBC, CRP and NLR in our study, which is indicative of the immune response towards the different types of antigens (cercariae, growing schistosomula, adult worms and eggs) exposed to the host body. The immune response to schistosomiasis is characterized by the formation of granulomas that consists of immune cells such as eosinophils and lymphocytes around egg antigens. Comparable results have been reported were there was increased WBC (59–61) IL-6 and TNF-α (62–64) in population groups with schistosomiasis in comparison to those without. Contrary to other studies we did not observe significant differences in neutrophils and lymphocytes counts between infected and uninfected populations (60, 65).

Uninfected PSAC were observed to have significantly higher concentration levels of IL-10 than infected PSAC and these results are comparable to studies that have reported on the presence of lowIL-10 levels in children with soil-transmitted helminths which could be due to the acute nature of *S. haematobium* infections in PSAC (66). Chronic infection would be required to induce high IL-10 immune responses.

We acknowledge the limitations of our study which include the inability of our results to infer casual relationships between the inflammatory biomarkers, schistosomiasis and cognitive performance. Our study findings might also differ with different ethnicity groups as inflammatory biomarkers such as cytokines have been reported to vary by race and there could have been the presence of other infectious diseases such as HIV which were not screened for in our study population (67). Nonetheless, our findings are indicative of the negative association of systemic inflammation on the cognitive performance of PSAC with schistosomiasis.

## Conclusion

In this study, high levels of inflammatory biomarkers (TNF-α, IL-6, IL-10, CRP, HGB, WBC, neutrophils and lymphocytes) were significantly correlated to lower cognitive performance in some of the Griffiths III cognitive domains (Foundations of Learning, Language and Communication, Eye-Hand-Coordination and General Development Domain). *Schistosoma haematobium* infections had a negative impact on the cognitive performance of PSAC in the Foundations of Learning, Eye-Hand-Coordination and General Development Domain. As this is the first study assessing the association of systemic inflammation with reduced cognitive performance in PSAC with schistosomiasis, we recommend further research to determine the relationships between inflammation and cognitive performance in diverse study populations groups across different ethnicities that have schistosome infections.

## Data availability statement

The raw data supporting the conclusions of this article will be made available by the authors, without undue reservation.

## Ethics statement

The studies involving human participants were reviewed and approved by Medical Research Council of Zimbabwe. Written informed consent to participate in this study was provided by the participants’ legal guardian/next of kin.

## Author contributions

Conceptualization of the study, MK, SM, FM, DC and TM. Parasitological analysis, MK, TM-J, AV, LJ and HM. Psychological clinical examination, AM, EM and BD-M. Serology lab work, MK. Data analysis, MK and RM. Manuscript writing, MK. Supervision, SM and TM. All authors contributed to the article and approved the submitted version.
